# Supplementary material for: Human MAIT cell cytolytic effector proteins synergize to overcome carbapenem resistance in Escherichia coli
Source: PLoS Biol. 2020 Jun 8;18(6):e3000644. doi: 10.1371/journal.pbio.3000644 (PMC7302869; doi:10.1371/journal.pbio.3000644)
Supplement: S1 Text — (DOCX) [file pbio.3000644.s002.docx]

**S1 Text. Additional Material and Methods**

**Additional information on bacterial cultures**

*E. coli* identification and antibiotic resistance profile was performed as described [1]. Briefly, genus identity was determined using VITEK GNI + cards (bioMérieux, Hazelwood, MO, USA). Antibiotic susceptibilities were determined using commercial microbroth dilution panels (Trek Diagnostics, East Grinstead, UK), performed according to manufacturer’s recommendations. *In silico* analysis of the draft genomes obtained from whole genome sequencing using the multilocus sequence typing (MLST) database (<http://pubmlst.org/>) was conducted to determine the sequence types. Carbapenemases and plasmid-mediated extended-spectrum beta-lactamases (ESBLs) and AmpCs were identified using the ResFinder tool (<https://cge.cbs.dtu.dk/services/ResFinder/>) [2]. The presence of efflux pumps was determined using the efflux pump inhibitor phenyl-arginine-beta-naphthylamide (PABN) (50 μg/mL), in accordance to a previously published protocol [3]. Levofloxacin MICs that were 4-fold lower in the presence of an efflux pump inhibitor were considered to be indicative of an elevated efflux mechanism [3].

**Microscopy**

HeLa cells were plated onto an 8-well Nunc Lab-Tek II Chamber Slide system (ThermoFisher Scientific) and incubated overnight at 37^0^C/5% CO_2_ in complete RPMI medium, then washed extensively to remove antibiotics and cultured in ASF-RPMI. Overnight *E. coli* strain EC120S subcultures were labelled with pHRodo Red (Thermo Fisher Scientific Life Sciences) as previously described [4], but without methanol fixation. HeLa cells were then infected with live, pHRodo Red-labelled *E. coli* strain EC120S for 3 h followed by removal of extracellular bacteria with high-dose gentamicin as described above. CTV-labelled MAIT cells were then added at a 2:1 E:T ratio. Apoptosis of target cells was determined by adding the fluorescent inhibitor of caspases (FLICA) reagent (Vybrant FAM Poly Caspases Assay Kit; Thermo Fisher Scientific) at a final concentration of 0.2% (v/v) to the MAIT-HeLa cell culture at the beginning of the assay as previously described [4]. Samples were imaged with a Nikon Ti2-D-PD microscope at 40x objective using the NIS-Elements BR v. 5.20.00 software (both from Nikon Instruments Inc.), with DAPI (for CTV), GFP (for FLICA), and mCherry (for pHrodo Red) filters. Microscopy data were then processed using Fiji image-processing software ([https://fiji.sc](https://fiji.sc/))

**Assessment of *E. coli* internalisation by target cells**

To determine *E. coli* internalisation by target cells (HeLa cells, A549 cells, or PBMCs), *E. coli* were labelled with pHRodo Red (Thermo Fisher Scientific Life Sciences) as previously described [4]. Labelled *E. coli* were then fed to the target cells for 3 h at 37^o^C, or on ice as negative controls for internalisation.

**Assessment of riboflavin synthesis competence of the clinical *E. coli* strains**

In selected experiments, 10^5^ CFU/mL *E. coli* were grown overnight at 37°C in Riboflavin Assay Medium (RAM; BD Biosciences) in the presence of 3 μg/mL riboflavin dissolved in acetonitrile (Honeywell), or in solvent control alone. *RibA*^-^ BSV18 cultures were supplemented with 20 μg/mL riboflavin (Sigma-Aldrich) [5]. Growth curves were monitored by reading absorbance at 600 nm in a microplate reader with discontinuous shaking for 16-18 h at 37^o^C (Cytation 5, BioTek Instruments).

***RibA* detection**

Bacteria were grown for 3h in LB medium. The bacterial pellet was frozen at -80°C until RNA isolation. Bacterial RNA was isolated with RNeasy Mini kit (Qiagen) according to manufacturer’s instructions. RNA was stored at -20°C until further use. After a step of DNA deactivation using Ambion DNase I RNase-free (Invitrogen), cDNA synthesis was done using the SuperScript VILO TM (Invitrogen). Primer set for *GyrA*: forward 5’-GTACGTTCATGGCGTAAAGT-3’, reverse 5’-CCGGTCAACATTGAGGAAGA-3’. Primer set for *RibA*: forward 5’-CCTAACTGGTTAGCCTC-3’, reverse 5’-CGGCATTGACGCAAATTGC-3’ (Eurofins Genomics). The real-time PCR amplification was performed with Quantitect SYBR Green PCR kit (Qiagen) using CFX96 Touch Real-Time PCR Detection System (Biorad). The levels of *ribA* was normalised over the *gyrA* levels using the Ct methods.

**Determination of bacterial growth curves and lag-phase**

Overnight bacteria subcultures were washed with PBS and resuspended in MAIT cell or control supernatants at 10^5^ CFU/mL with or without imipenem supplementation as indicated. Bacterial suspensions were incubated at 37^0^C in flat-bottom 96-well plates in a microplate reader with absorbance at 600 nm and discontinuous shaking at 250 rpm for 5 seconds done every 10 min for 18 h at 37^o^C (Cytation 5, BioTek Instruments). The lag-phase periods of the bacteria were extrapolated from the resulting growth curves by the in-build Gen5 v. 3.03 data analysis software (BioTek Instruments). If there were no detectable growth by the end of the 18 h-incubation, then the lag-phase periods were indicated as 18 h.

**Additional** **MAIT cell secretome antimicrobial activity assay**

Ertapenem was purchased from Sigma-Aldrich and meropenem was provided by the Singapore General Hospital pharmacy. In selected experiments, the 5-OP-RU-pulsed 293T-hMR1 cell +/- MAIT cell co-culture were treated with 5 mM EGTA supplemented with 1 mM MgCl_2_, 10 μM CAS-BIND Pro Pan Caspase Inhibitor, or 10 μg/mL of anti-IFNγ, anti-TNF, anti-IL-17A, or IgG1 isotype control during the 24 h co-culture. The EGTA-treated supernatants were re-supplemented with 5 mM CaCl_2_  and 1 mM MgCl_2_ just prior to the antimicrobial assay. To inhibit GrzB activity, 100 μM of the Granzyme B inhibitor II Ac-IETD-CHO or vehicle was added into the the supernatants immediately prior to the antimicrobial assay.

**Granulysin and perforin depletion from MAIT cell supernatants**

Purified mAbs against Gnly (clones DH2 and DH10; Biolegend), Prf (clone dG9; Biolegend), or IgG1 isotype control (clone MOPC-21, Biolegend) were added into the MAIT cell supernatants at 20 μg/mL each and incubated overnight at 4^o^C with continuous mixing. 30 μL of 50% (v/v) protein G sepharose beads slurry (GE Healthcare) were added and further incubated for 1 h at 4^o^C with continuous mixing. Depleted or mock-depleted (isotype-treated) supernatants were clarified by centrifugation and immediately used for the antimicrobial activity assay as described, or snap-frozen in liquid nitrogen and stored in -80^o^C for supernatant analyses.

**References**

1. Teo JQ, Chang CW, Leck H, Tang CY, Lee SJ, Cai Y, et al. Risk factors and outcomes associated with the isolation of polymyxin B and carbapenem-resistant Enterobacteriaceae spp.: A case-control study. Int J Antimicrob Agents. 2019;53(5):657-62. Epub 2019/03/19. doi: 10.1016/j.ijantimicag.2019.03.011. PubMed PMID: 30880229.

2. Zankari E, Hasman H, Cosentino S, Vestergaard M, Rasmussen S, Lund O, et al. Identification of acquired antimicrobial resistance genes. J Antimicrob Chemother. 2012;67(11):2640-4. Epub 2012/07/12. doi: 10.1093/jac/dks261. PubMed PMID: 22782487; PubMed Central PMCID: PMCPMC3468078.

3. Davies TA, Marie Queenan A, Morrow BJ, Shang W, Amsler K, He W, et al. Longitudinal survey of carbapenem resistance and resistance mechanisms in Enterobacteriaceae and non-fermenters from the USA in 2007-09. J Antimicrob Chemother. 2011;66(10):2298-307. Epub 2011/07/22. doi: 10.1093/jac/dkr290. PubMed PMID: 21775338.

4. Dias J, Sobkowiak MJ, Sandberg JK, Leeansyah E. Human MAIT-cell responses to Escherichia coli: activation, cytokine production, proliferation, and cytotoxicity. J Leukoc Biol. 2016;100(1):233-40. doi: 10.1189/jlb.4TA0815-391RR. PubMed PMID: 27034405; PubMed Central PMCID: PMC4946616.

5. Dias J, Boulouis C, Gorin JB, van den Biggelaar R, Lal KG, Gibbs A, et al. The CD4(-)CD8(-) MAIT cell subpopulation is a functionally distinct subset developmentally related to the main CD8(+) MAIT cell pool. Proceed Natl Acad Sci USA. 2018;115(49):E11513-E22. Epub 2018/11/18. doi: 10.1073/pnas.1812273115. PubMed PMID: 30442667; PubMed Central PMCID: PMCPMC6298106.

6. Roederer M, Nozzi JL, Nason MC. SPICE: exploration and analysis of post-cytometric complex multivariate datasets. Cytometry A. 2011;79(2):167-74. Epub 2011/01/26. doi: 10.1002/cyto.a.21015. PubMed PMID: 21265010; PubMed Central PMCID: PMCPMC3072288.
