# Supplementary material for: Human MAIT cell cytolytic effector proteins synergize to overcome carbapenem resistance in Escherichia coli
Source: PLoS Biol. 2020 Jun 8;18(6):e3000644. doi: 10.1371/journal.pbio.3000644 (PMC7302869; doi:10.1371/journal.pbio.3000644)
Supplement: S1 Table — (DOCX) [file pbio.3000644.s003.docx]

**S1 Table. The *E. coli* clinical isolates used in this study**

| **ID** | **Site of culture** | **Carbapenemase**  **(WGS)** | **Major ESBL**  **(WGS)** | **Efflux protein** | ***mcr-1*** | **MLST** |
| --- | --- | --- | --- | --- | --- | --- |
| EC234 | Blood | NDM-7  (Ambler class B) | CTXM-15, CMY-42, OXA-1 | – | – | 205 |
| EC241 | Sputum | KPC-2  (Ambler class A) | OXA-1, TEM-1B | + | – | 131 |
| EC362 | Sore tissue | KPC-2  (Ambler class A) | TEM-1B, TEM-215 | ND | + | 224 |
| EC385 | Blood | OXA-48  (Ambler class D) | CTXM-55, TEM-1B | – | – | 101 |
| EC120 | Blood | Non-CRE ctrl | ND | ND | ND | ND |

AmpC, ampicillin resistance gene group C; CMY, cephamycinase; CRE, carbapenem-resistant Enterobacteriaceae; CTXM, active on cefotaxime–Munich; ESBL, extended-spectrum β-lactamase; KPC, *Klebsiella pneumoniae* carbapenemase; *mcr-1*, mobilised colistin resistance; MLST, multilocus sequence typing; NDM, New Delhi metallo-β-lactamase; OMP, outer membrane protein; OXA, oxacillinase-group; TEM, Temoniera; WGS, whole genome sequencing. ND, no data.
