# Supplementary material for: Human MAIT cell cytolytic effector proteins synergize to overcome carbapenem resistance in Escherichia coli
Source: PLoS Biol. 2020 Jun 8;18(6):e3000644. doi: 10.1371/journal.pbio.3000644 (PMC7302869; doi:10.1371/journal.pbio.3000644)
Supplement: S2 Table — MIC, minimum inhibitory concentrations. (DOCX) [file pbio.3000644.s004.docx]

**S2 Table. The minimum inhibitory concentrations (MIC) of the *E. coli* clinical isolates**

| **ID** | **LVX** | **ETP** | **IPM** | **MEM** | **DOR** | **ATM** | **TZP** | **FEP** | **AMK** | **CST** | **TGC** | **GEN^#^** |
| --- | --- | --- | --- | --- | --- | --- | --- | --- | --- | --- | --- | --- |
| EC234 | ≥32 | ≥32 | ≥32 | ≥32 | ≥32 | ≥64 | ≥128 | ≥64 | 16 | 1 | 0.5 | S |
| EC241 | ≥32 | ≥32 | 8 | 16 | 8 | ≥64 | ≥128 | ≥64 | 16 | 1 | **≤**0.25 | S |
| EC362 | ≥32 | ≥32 | 8 | 16 | 8 | ≥64 | ≥128 | ≥64 | **≤**4 | 4 | 0.5 | S |
| EC385 | ≥32 | ≥32 | 2 | 0.5 | 2 | ≥64 | ≥128 | ≥64 | **≤**4 | 1 | 0.5 | S |
| EC120 | **≤**0.25 | **≤**0.125 | **≤**0.25 | **≤**0.25 | **≤**0.25 | **≤**0. 5 | **≤**0.25 | **≤**1 | **≤**4 | 1 | **≤**0.25 | S |

The MIC The MIC (μg/mL) was performed using broth dilution method. Antimicrobial breakpoint to interpret as susceptible (S), susceptible dependent on dose, or resistant (R) was determined based on the European Committee on Antimicrobial Susceptibility Testing (EUCAST) 2018 guidelines. AMK, amikacin; ATM, aztreonam; CST, colistin; DOR, doripenem; ETP, ertapenem; FEP, cefepime; GEN, gentamicin; IPM, imipenem; LVX, levofloxacin; MEM, meropenem; TGC, tigecycline; TZP, piperacillin-tazobactam. ^#^Kirby-Bauer disk diffusion test. The carbapenems are shaded.
