# Supplementary material for: Human MAIT cell cytolytic effector proteins synergize to overcome carbapenem resistance in Escherichia coli
Source: PLoS Biol. 2020 Jun 8;18(6):e3000644. doi: 10.1371/journal.pbio.3000644 (PMC7302869; doi:10.1371/journal.pbio.3000644)
Supplement: S3 Table — (DOCX) [file pbio.3000644.s005.docx]

**S3 Table.** Flow cytometry-based antibodies and reagents used in the study

| **Antibody name** | **Clone** | **Antibody source** |
| --- | --- | --- |
| anti-active Caspase 3 Brilliant Violet (BV) 650, PE  anti-CD3 FITC, Alexa Fluor (AF) 700, PE-CF594  anti-CD45 BUV395  anti-CD69 AF700  anti-CD103 BV605  anti-CD107a BUV395  anti-CD161 PECy5, FITC  anti-GrzB AF700, BV421  anti-IFNγ APC | C92-605  UCHT1  HI30  FN50  Ber-ACT8  H4A3  DX12  GB11  B27 | **BD Biosciences** |
|  |  |  |
| anti-CD3 BV650 | OKT3 | **BioLegend** |
| anti-CD161 PE | HP-3G10 |  |
| anti-Gnly PE, AF647 | DH2 |  |
| anti-GrzA AF700 | CB9 |  |
| anti-GrzB B FITC  anti-IL-17A BV785 | GB11  BL168 |  |
| anti-Prf BV421, PECy7  anti-TNF BV711 | B-D48  MAb11 |  |
| anti-Vα7.2 BV605, PE, PECy7  LEGENDplex Human CD8/NK Panel (13-plex) | 3C10 |  |
| CellTrace Violet Cell Proliferation Kit  Vybrant FAM Poly Caspases (FLICA) Assay Kit  LIVE/DEAD® Fixable Aqua, Near-IR Dead Cell Stain Kits  pHrodo Red, succinimidyl ester  SYTO 62 Red Fluorescent Nucleic Acid Stain  SYTOX Green Nucleic Acid Stain |  | **Invitrogen/Life Technologies/ Thermo Fisher** |
| GranToxiLux PLUS! |  | **OncoImmunin Inc.** |
| Human MR1 5-OP-RU PE, BV421, AF680  Human MR1 6-FP PE, AF680 | MR1 tetramers | **The NIH Tetramer Core Facility at Emory University** |
