# Supplementary figures and images for: Human MAIT cell cytolytic effector proteins synergize to overcome carbapenem resistance in Escherichia coli
Source: PLoS Biol. 2020 Jun 8;18(6):e3000644. doi: 10.1371/journal.pbio.3000644 (PMC7302869; doi:10.1371/journal.pbio.3000644)

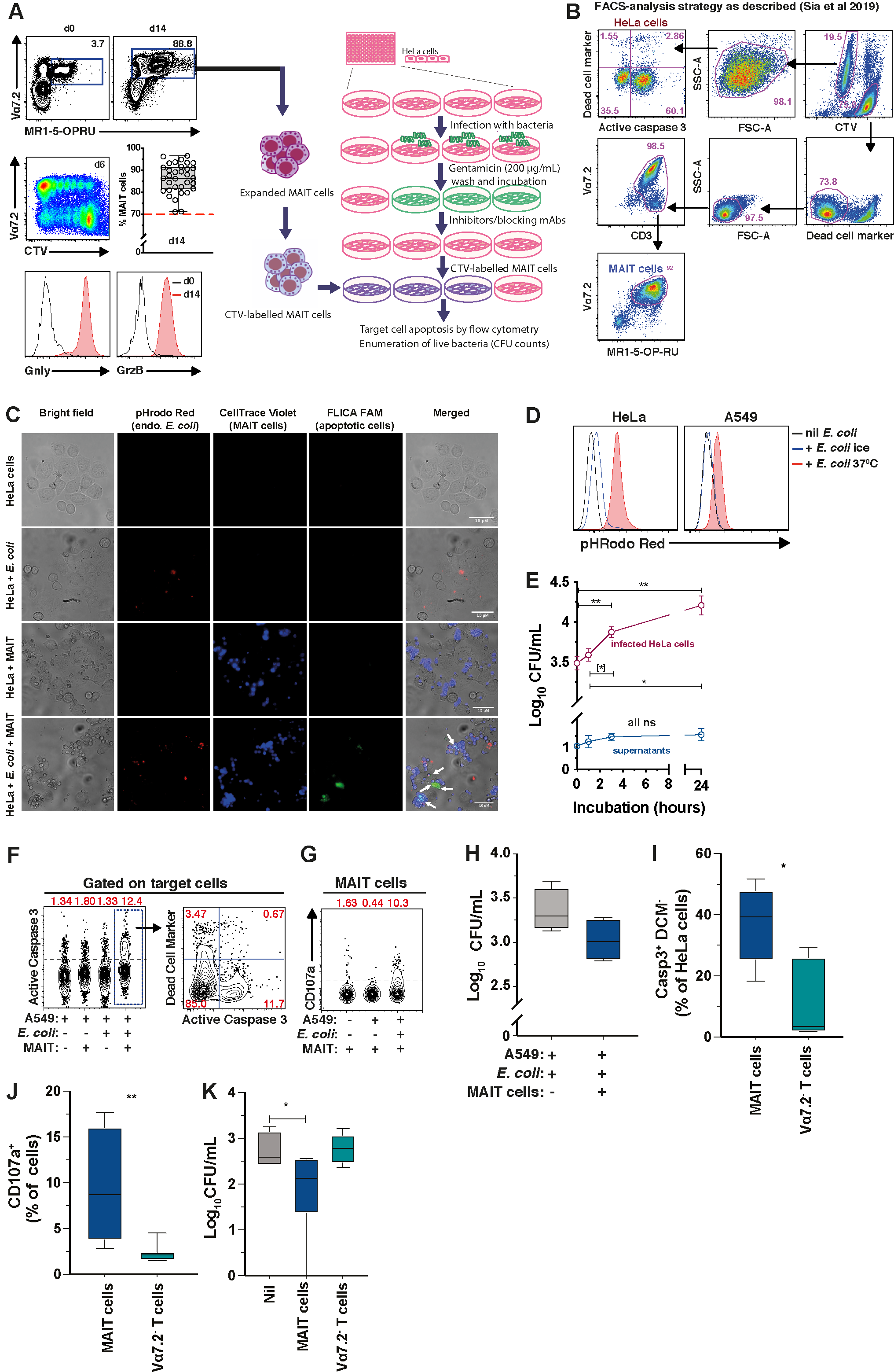

Supplement: S1 Fig — (A) An illustration of the protocol for the killing assay of E. coli–infected HeLa cells by MAIT cells and MAIT cell antimicrobial activity, along with a representative FACS plot of MAIT cell proliferation on day 6 using CTV dilution assay and % MAIT cells (left middle panels) and expression of Gnly and GrzB (left bottom panels) following 14 d of in vitro expansion, and (B) the gating strategy for the flow cytometry analyses. (C) Representative fluorescence microscopy images of MAIT cells (blue)-mediated killing of HeLa cells (green) following internalization of pHrodo red-labeled live E. coli EC120S (red) by HeLa cells depicted by white arrows (n = 3 independent experiments). (D) pHRodo-labeled E. coli strain EC120S uptake by HeLa and A549 cells for 3 h on ice or at 37 °C (n = 3 independent experiments). (E) Bacterial loads of E. coli strain EC120S over 24 h at 37 °C inside the infected HeLa cells and in supernatants in the presence of low concentration (20 μg/mL) of gentamicin (n = 4–11 per time point). (F) Representative flow cytometry staining of active Casp3 and amine-reactive dead cell marker in A549 cells alone, A549 cells infected with E. coli EC120S, or A549 cells co-cultured with MAIT cells with or without E. coli EC120S for 24 h. (G) Representative flow cytometry plot of CD107a/degranulation in MAIT cells alone, or co-cultured with A549 cells with or without E. coli EC120S. (H) Bacterial counts in E. coli EC120S-infected A549 cells co-cultured with or without MAIT cells for 24 h (n = 4). (I, J, K) Apoptosis of HeLa cells (I), degranulation of effector cells (J), and bacterial counts (K) in the HeLa-MAIT or HeLa-Vα7.2− T cells co-culture with or without E. coli EC120S (n = 5–6 in panels I and K and n = 8 in panel J). Data presented as line with error bars represent the mean and standard error. Box and whisker plots show median, the 10th to 90th percentile, and the interquartile range. Statistical significance was determined using mixed-effects analysis fol [file pbio.3000644.s006.tif]

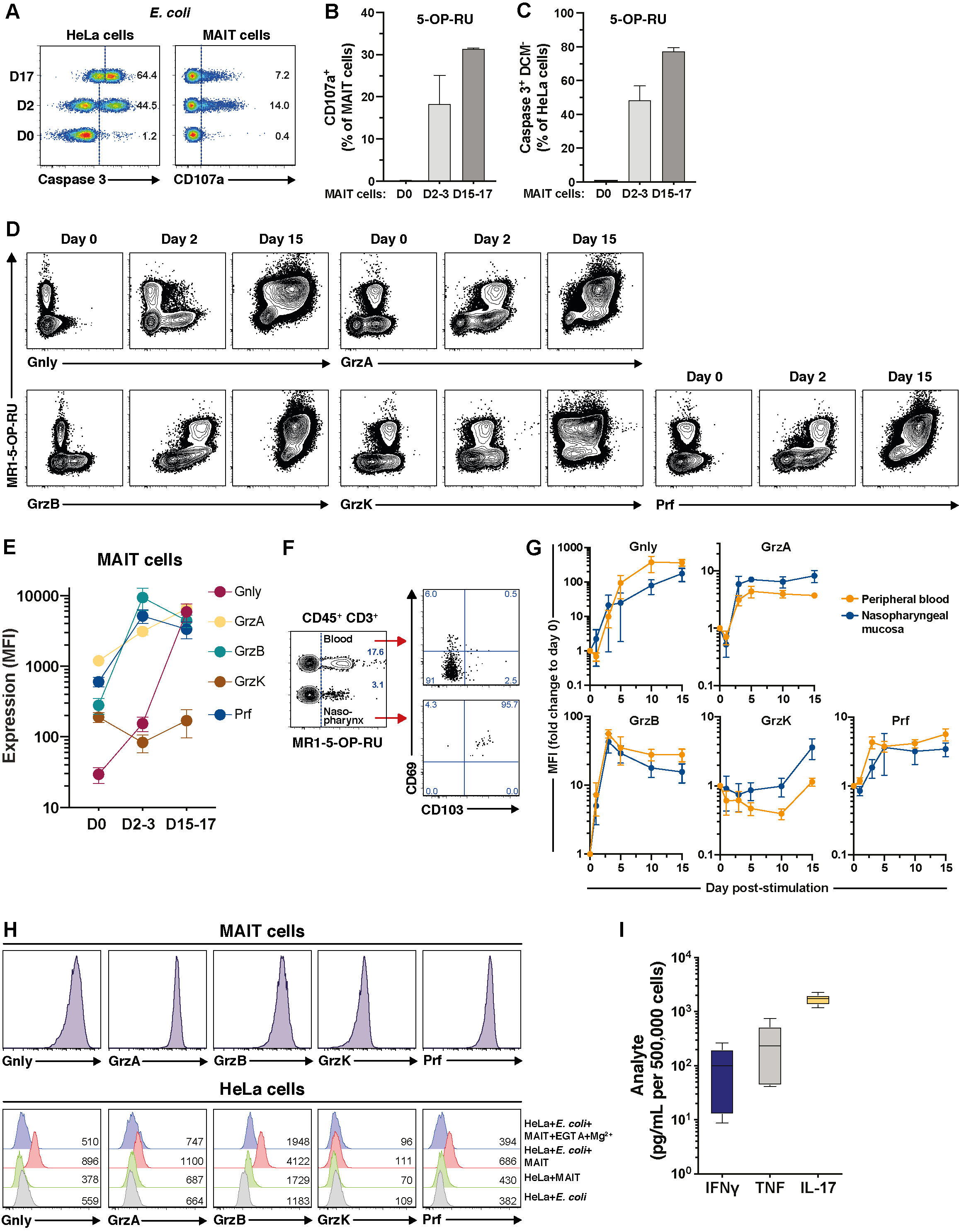

Supplement: S2 Fig — (A) Representative flow cytometry staining of Casp3 expression in HeLa cells and CD107a degranulation in MAIT cells stimulated with E. coli EC120S for 24 h using MAIT cells from D0, D2, and D15 after expansion (protocol 2). (B, C) Casp3 expression in HeLa cells and CD107a degranulation in MAIT cells stimulated with the MR1 ligand 5-OP-RU for 24 h using MAIT cells from D0 and D2 and D15 after expansion (all n = 4). (D, E) Representative flow cytometry data (D) and combined data (E) of GrzA, GrzB, GrzK, Gnly, and Prf (n = 4–10) levels (MFI) in MAIT cells over the course of the in vitro expansion. (F) Identification of matched PB and tissue-resident MAIT cells from the NP mucosae of 3 healthy individuals undergoing nasal polyp removal. (G) Relative expression levels (fold change of MFI to D0) of cytolytic proteins expressed by matched PB and NP MAIT cells at baseline and at various time points following in vitro expansion (n = 3–4). (H) Detection of cytolytic protein contents in the effector MAIT cells and target E. coli EC120S-infected HeLa cells following 3 h co-culture with MAIT cells in the presence or absence of EGTA + Mg2+. Representative histograms from at least 2 independent MAIT cell donors are shown. (I) Levels of cytokines in the supernatants following MAIT cell co-culture with E. coli EC120S-infected HeLa cells for 3 h (n = 6). Data presented as line or bar graphs with error bars represent the mean and standard error. Box and whisker plots show median, the 10th to 90th percentile, and the interquartile range. The underlying data of this figure can be found in S1 Data. Casp, caspase; D, day; Gnly, granulysin; Grz, Granzyme; IFNγ, interferon-γ; IL-17A, interleukin-17A; MAIT, Mucosa-associated invariant T; MFI, mean fluorescence intensity; MR1, MHC-Ib-related protein; NP, nasopharyngeal; PB, peripheral blood; Prf, perforin; 5-OP-RU, 5-(2-oxopropylideneamino)-6-D-ribitylaminouracil. (TIF) [file pbio.3000644.s007.tif]

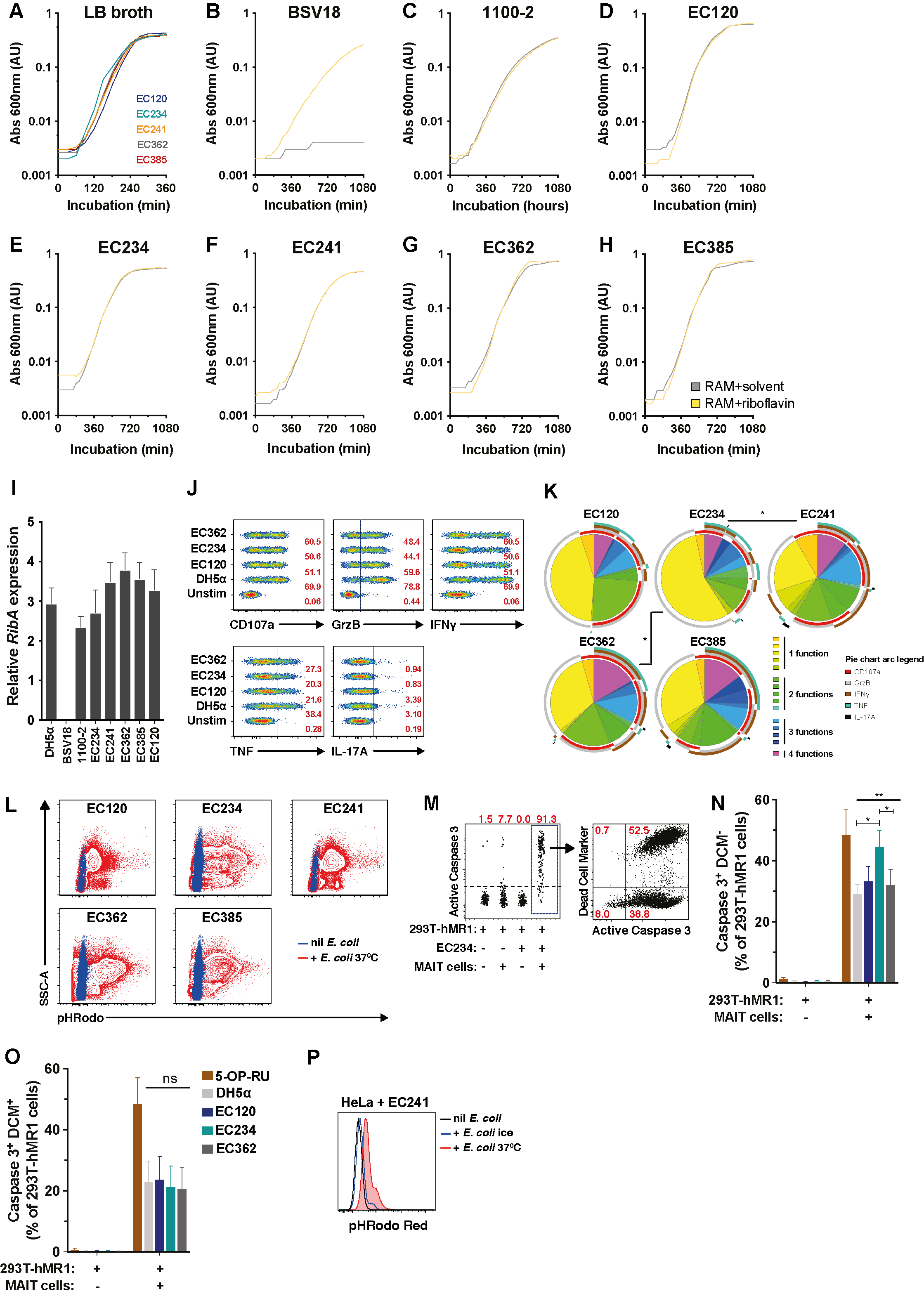

Supplement: S3 Fig — (A–H) Growth curve of the E. coli strains BSV18 (RibA−), 1100–2 (RibA+ isogenic strain of BSV18), EC120S, EC234, EC241, EC362, and EC385 in LB or in riboflavin-deficient medium with supplemental riboflavin or acetonitrile solvent control (n = 3). (I) Relative RNA expression of RibA of the indicated E. coli (n = 3 independent experiments). (J) Representative flow cytometry plots of degranulation (CD107a) and production of GrzB, IFNγ, TNF, and IL-17A by MAIT cells following stimulation of PBMCs with formaldehyde-fixed E. coli strains DH5α, EC120S, EC234, and EC362. (K) Polyfunctional profile of MAIT cell responses against the indicated E. coli strains presented in pie charts (n > 5). Comparison of the pie chart distributions was performed using a partial permutation test and performed using SPICE version 5.1, downloaded from http://exon.niaid.nih.gov [6] (L) Bacterial uptake by PBMC (n = 3) in the presence of pHrodo-labeled E. coli strains as indicated for 3 h on ice or at 37 °C. (M) Representative flow cytometry plots of Casp3 activation and apoptosis in 293T-hMR1 cells alone, 293T-hMR1 cells infected with EC234, or co-culture with MAIT cells with or without EC234 for 24 h. (N, O) Casp3 activation and apoptosis in 293T-hMR1 cells alone or co-cultured with MAIT cells in the presence of 5-OP-RU or E. coli strains DH5α, EC120S, EC234, or EC362 (n = 7–9). MAIT cells were expanded polyclonally for 7 d as described. (P) Bacterial uptake by HeLa cells (n = 3 independent experiments) in the presence of pHrodo-labeled E. coli EC241 for 3 h on ice or at 37 °C. Data presented as line graphs represent the mean, and bar graphs with error bars represent the mean and standard error. Box and whisker plots show median, the 10th to 90th percentile, and the interquartile range. Statistical significance was determined using the Friedman test (N, O) followed by Dunn’s multiple comparison test. **p < 0.01, *p < 0.05. The underlying data of this figure can be found in S1 Data. Abs, absorba [file pbio.3000644.s008.tif]

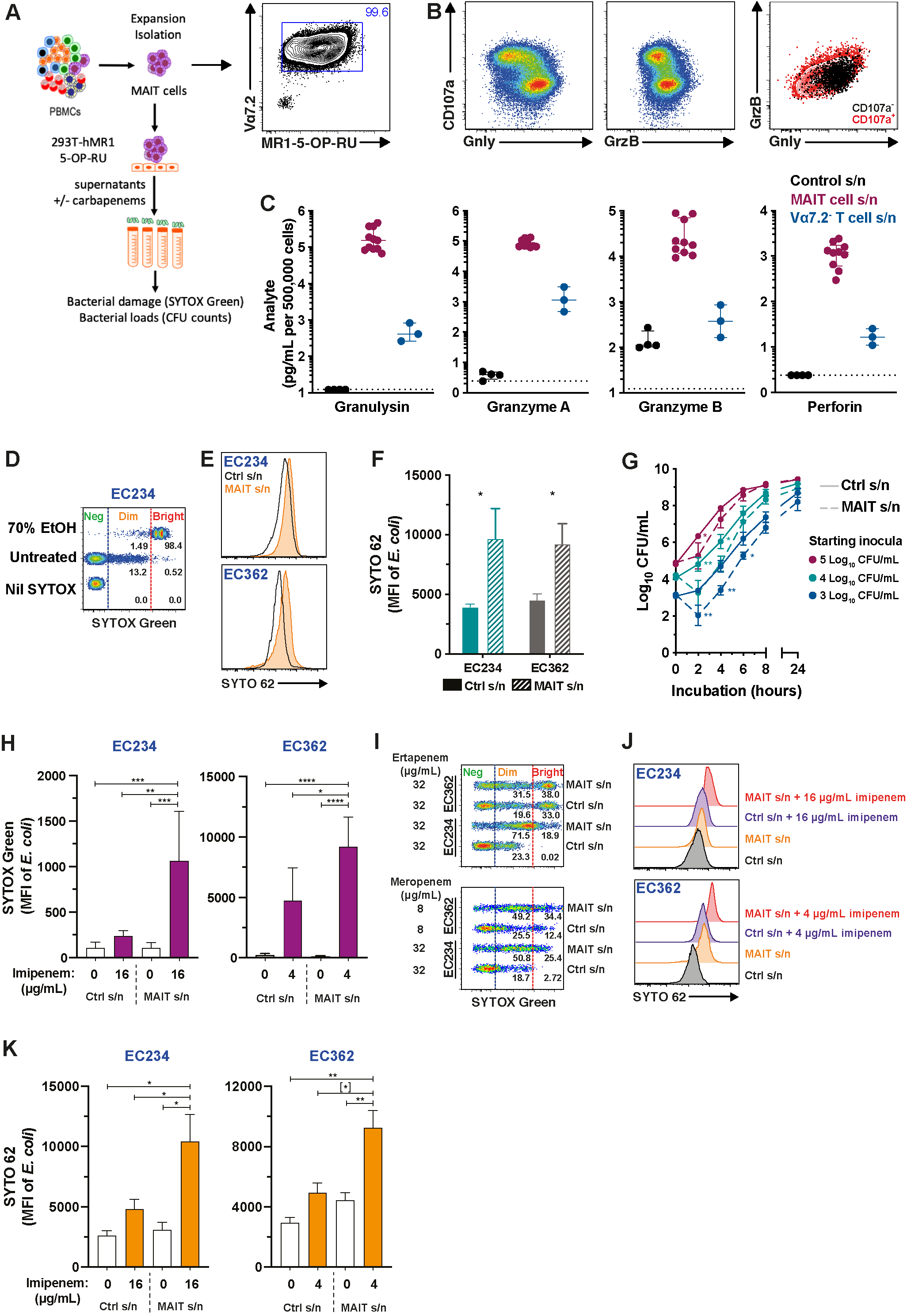

Supplement: S4 Fig — (A) An illustration of the protocol to test the antimicrobial activity of the MAIT cell secretomes using flow cytometry stainings of the bacteria and live bacterial counts using the traditional agar plating method. (B) Representative flow cytometry plots of MAIT cell degranulation (CD107a) and expression of Gnly and GrzB following stimulation with 5-OP-RU-pulsed 293T-hMR1 cells. (C) Concentration of Gnly, GrzA, GrzB, and Prf secreted by MAIT cells and Va7.2− T cells stimulated with 5-OP-RU-pulsed 293T-hMR1 cells. (D) Representative flow cytometry plots of SYTOX Green expression in E. coli strain EC234 untreated (live) or killed with 70% (v/v) ethanol. (E, F) Representative flow cytometry histograms (E) and combined data (F) of SYTO 62 expression in E. coli strains EC234 and EC362 in the presence of MAIT cell or control supernatants (n = 6). (G) Live E. coli EC362 bacterial counts over 24 h in the presence of control or supernatants prepared from MAIT cells expanded polyclonally for 7 d as described (n = 4–6). (H) SYTOX Green MFI in EC234 and EC362 in the presence of MAIT cell or control supernatants and with or without imipenem for 2 h (n = 8–10). (I, J, K) Representative flow cytometry plots of SYTOX Green staining (I), and histograms (J) and combined data (K) of SYTO 62 staining and levels on strains EC234 and EC362 following 2 h incubation with MAIT cell or control supernatants with or without imipenem (n = 6–8), ertapenem (n = 3 for both strains), or meropenem (n = 3 for both strains). Statistical significance was calculated using the paired t test (F), two-way ANOVA or mixed-effects analysis with Sidak’s multiple comparison test (G), and RM one-way ANOVA with Dunnett’s multiple comparisons test (H, J). The bar graphs and error bars represent the mean and standard error, whereas scatter plots show median and the IQR. ****p < 0.0001, ***p < 0.001, **p < 0.01, *p < 0.05, [*]p < 0.1. The underlying data of this figure can be found in S1 Data. CFU, colony-forming un [file pbio.3000644.s009.tif]

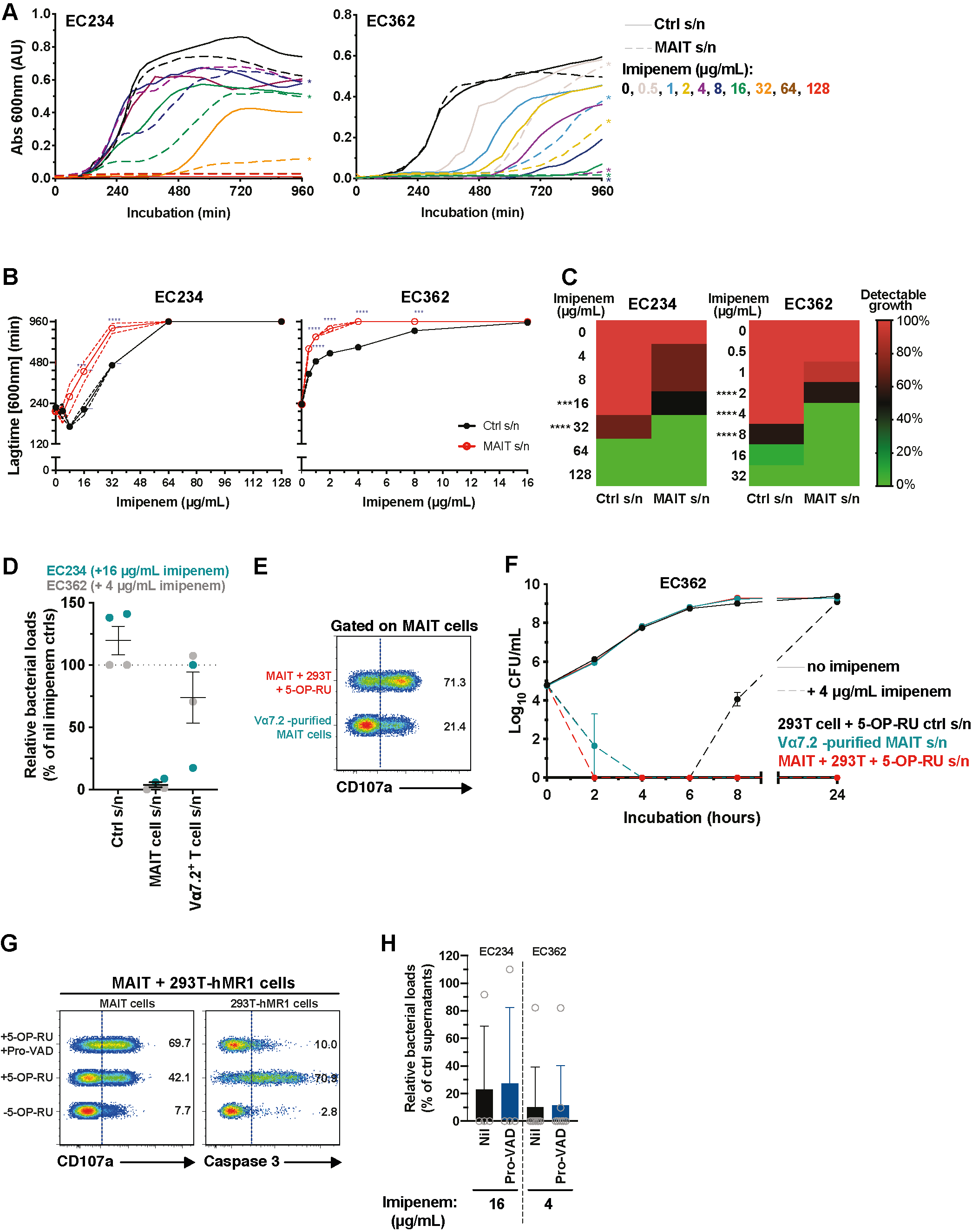

Supplement: S5 Fig — (A, B) Growth curves (A) and the lag phase (B) of the E. coli strains EC234 and EC362 in presence of MAIT cell or control supernatants and at the indicated concentration of imipenem (n = 2–14, both strains). (C) Heat map of detectable growth of strains EC234 and EC362 after 18 h incubation in MAIT cell or control supernatants and at the indicated concentration of imipenem (n = 10 [EC234], 11 [EC362]). (D) Relative bacterial loads of strains EC234 and EC362 in presence of supernatants derived from 5-OP-RU-pulsed 293T cells alone (control), or following co-culture with MAIT cells or Vα7.2− T cells. (E, F) Representative flow cytometry plots of degranulation (CD107a) (E) by Vα7.2-bead-purified and -activated MAIT cells without further co-culture with Ag-presenting cells and that of bulk MAIT cells co-cultured with 5-OP-RU-pulsed 293T-hMR1 cells. E. coli strain EC362 was then incubated with clarified supernatants that were collected 24 h after co-culture in the presence of imipenem (F) and the bacterial loads were determined over time (n = 2). (G) Representative flow cytometry plots of degranulation by MAIT cells and Casp3 activation in 293T-hMR1 cells in the co-culture untreated or in the presence of 5-OP-RU with or without the pan-Casp inhbitor Pro-VAD. (H) Relative bacterial loads of the strains EC234 or EC362 after 24 h incubation with imipenem-supplemented MAIT cell supernatants prepared in the presence or absence of Pro-VAD (n = 4 [EC234], 6 [EC362]). Significant differences between control and MAIT cell supernatants at indicated imipenem concentrations were calculated using mixed-effects analysis with Sidak’s multiple comparisons test (A–C). The heat map shows the mean, the lines of the growth lag-phase curves represent the mean, the bar graphs and error bars represent the mean and standard error, whereas box and whisker plots show median, the 10th to 90th percentile, and the IQR. The underlying data of this figure can be found in S1 Data. Abs, absorbance; AU, ar [file pbio.3000644.s010.tif]

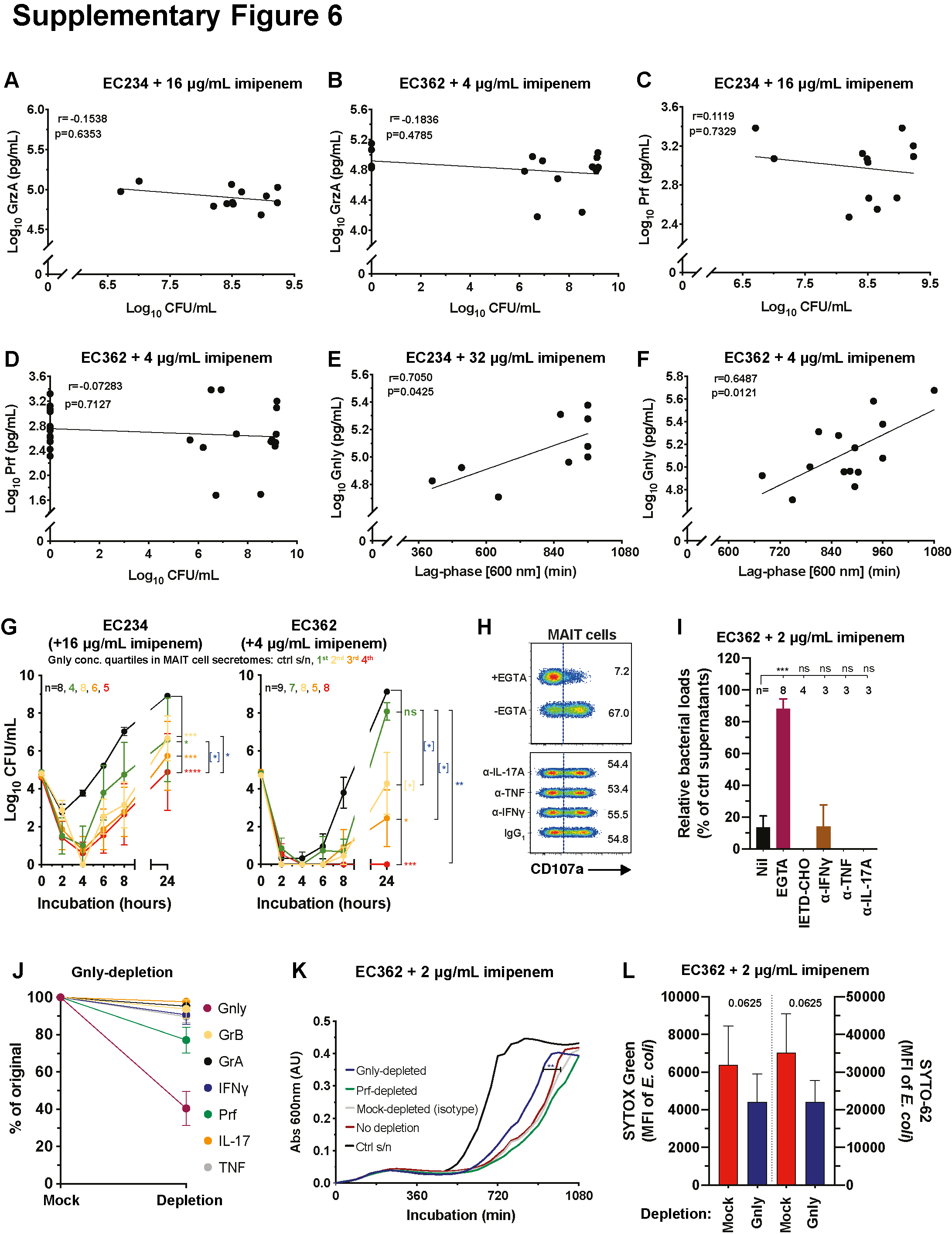

Supplement: S6 Fig — (A–F) Correlation between the concentration of GrzA (A, B), Prf (C, D) and Gnly (E, F) in the supernatant of MAIT cells stimulated with 5-OP-RU-pulsed 293T-hMR1 cells with the bacterial loads of E. coli EC234 (A, C) and EC362 (B, D), or with the lag-phase of strains EC234 (E) and EC362 (F) in the presence of the indicated concentration of imipenem (n = 12 in panels A and C; n = 17 in panel B; n = 28 in panel D; n = 9 in panel E; n = 14 in panel F). (G) Analyses of live bacterial counts of E. coli EC234 and EC362 based on the Gnly concentration levels following 24 h incubation in control or MAIT cell supernatants supplemented with imipenem. (H) Representative flow cytometry plots of MAIT cell degranulation following co-culture with 5-OP-RU-pulsed 293T-hMR1 cells in the presence or absence of EGTA, anti-IFNγ, anti-TNF, anti-IL-17A, or IgG1 isotype control. (I) The relative E. coli EC362 bacterial loads following 24 h incubation in MAIT cell supernatants to those of control supernatants (both supplemented with 2 μg/mL imipenem) spiked with the degranulation inhibitor EGTA, GrzB inhibitor IETD-CHO, and mAbs against IFNγ, -TNF, or -IL-17A. For EGTA and mAb/isotype control-treated supernatants, EGTA and respective mAbs were added during the 24 h MAIT cells + 293T-hMR1 cells + 5-OP-RU co-culture stage, then clarified supernatants were used for the antimicrobial activity assay as described. (J) Proportion of Gnly and other effector molecules remaining when compared to mock-depleted (IgG1 isotype-treated) supernatant following Gnly-specific depletion of MAIT cell supernatants (n = 3–4). (K) Growth curves of strain EC362 with 2 µg/mL of imipenem in the presence of control or MAIT cell supernatant, or in mock-, Gnly-, or Prf-depleted MAIT cell supernatants (n = 3). (L) SYTOX Green and SYTO 62 staining intensity (MFI) of strain EC362 following 2 h-treatment with 2 µg/mL of imipenem in the presence of mock- or Gnly-depleted MAIT cell supernatant (n = 5). Significant differences [file pbio.3000644.s011.tif]

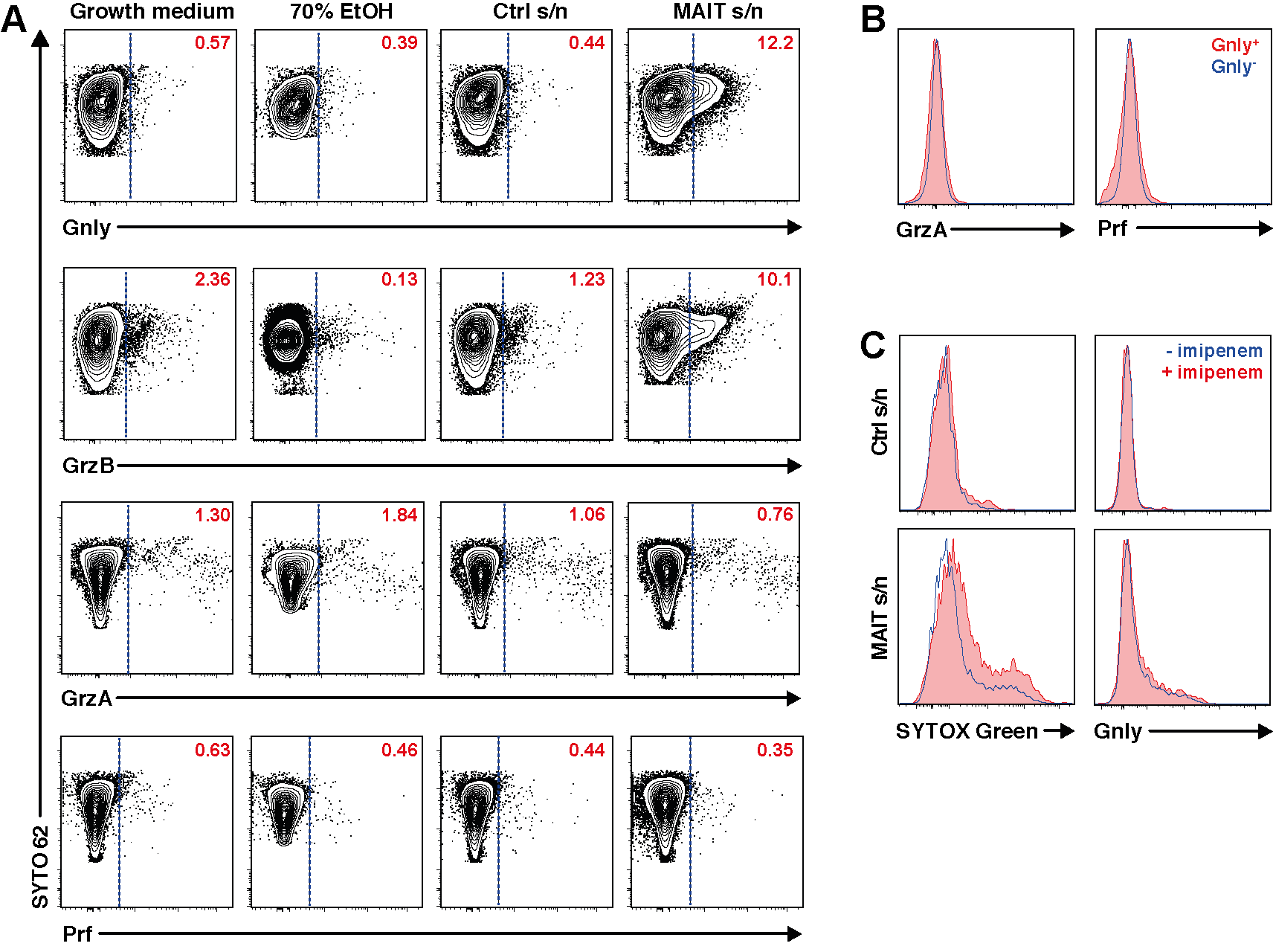

Supplement: S7 Fig — (A–C) Representative flow cytometry plots (A) and histograms (B) of cytolytic protein expression and (C) SYTOX Green staining intensity of E. coli strain EC362 treated with growth medium (LB), 70% ethanol, 293T-hMR1+5-OP-RU (control) supernatant, and 293T-hMR1+MAIT+5-OP-RU (MAIT) supernatant in the absence or presence of 2 μg/mL imipenem as indicated (n = 6). Ctrl, control; EtOH, ethanol; Gnly; granulysin; Grz, granzyme; LB, Luria (lysogeny) broth; MAIT, Mucosa-associated invariant T; Prf, perforin; s/n, supernatant; 293T-hMR1, 293T cells stably transfected with human MR1; 5-OP-RU, 5-(2-oxopropylideneamino)-6-D-ribitylaminouracil. (TIF) [file pbio.3000644.s012.tif]
