# Supplementary material for: Human MAIT cell cytolytic effector proteins synergize to overcome carbapenem resistance in Escherichia coli
Source: PLoS Biol. 2020 Jun 8;18(6):e3000644. doi: 10.1371/journal.pbio.3000644 (PMC7302869; doi:10.1371/journal.pbio.3000644)
Supplement: Attachment — Submitted filename: Boulouis.docx [file pbio.3000644.s013.docx]

In this manuscript, Boulouis and colleagues describe a possible mechanism underscoring the antimicrobial activity of MAIT cells, based on the cytolytic proteins granulysin and granzyme B, released by activated MAIT cells in an MR1 dependent manner.

The paper is well written, the experiments are well controlled and the data are convincing. This is an important study that extends our understanding of the biological functions of MAIT cells, and suggests way of potentially harnessing these cells for clinical benefit, during antibiotic resistant infections.

The main limitation of this study is that the antimicrobial effect of MAIT cell supernatants is only observed upon culturing the cells for two weeks, and although this is well discussed and acknowledged, it has not been investigated whether tissue resident MAIT cells (ie in the gastrointestinal tract) would express higher levels of granzyme B and granulysin or whether upon infection, in vivo, the kinetics of GrzB and Gly acquisition are faster.

I have a few minor comments:

1. The legend of Figure 1 G-J is not clear: the text talks about cells activated by 5OPRU or E coli but this is not clear from the graphs; furthermore, it is not clear how the heat map in panel J is derived: are these FACS data?
2. Figure S2E is misleading, if compared to S2D: although the colour scale reflects the MFI values, it gives the false impression that some markers are not expressed on D0, which is clearly not the case for Granzyme A and perforin. Why was Granzyme K not measured?
3. In Figure 1A and S2F it would be good to have the values of MAIT cell expression of the indicated proteins shown as additional overlay. Also, the authors should show the gating strategy used to discriminate between Hela and MAIT cells.
4. In Figure 3A, IL17 secretion by MAIT cells is very low and without negative controls (unpulsed target cells for example), should be interpreted with caution.
5. Figure 3: It is not clear why in panels C-E MR1 overexpressing 293T cells were used. The transition from 293T (panels C-E) to Hela is also confusing. Lastly, what is the difference between panels D and E?
6. Figure 4: in panel D, at 2 hours, the authors detect a significant difference in bacterial counts, upon exposure to activated MAIT supernatants. This, however, is not reflected in the time curves in panels G, H and in Figure S5A and C.
7. The text mentions (page 14) expression of GrzA and Prf in figure 5I, but this is only shown in S7A and B.
8. Figure S1A, is the y axis correct? Should it not be % of CD107+ cells, without MAIT?
9. Figure S3K, the colour legend is missing.
10. Colours in the growth curves (S5A, S6K) are often difficult to distinguish, perhaps a thicker line could be helpful.
11. Why the supernatant of 293T and 5OPRU (but no MAIT cells) has an anti-bacterial effect in Fig S5F?
12. The dual labelling of bacteria with Sytox green and Syto62 is very elegant, but from the text I had the impression bacteria were labelled with Syto62 before exposure to MAIT supernatants and carbapenem, hence it was not clear why MFI of Syto62 was also increasing. This was clear after reading the methods, so it would help if the main text or the legend also explained the experiment.
