# Supplementary material for: Human MAIT cell cytolytic effector proteins synergize to overcome carbapenem resistance in Escherichia coli
Source: PLoS Biol. 2020 Jun 8;18(6):e3000644. doi: 10.1371/journal.pbio.3000644 (PMC7302869; doi:10.1371/journal.pbio.3000644)
Supplement: Attachment — Submitted filename: Boulouis et al_PLOSBIO_rebuttal_final.docx [file pbio.3000644.s014.docx]

Dear Dr. Di Jiang,

We are very pleased that you are willing to consider a revised version of our manuscript PBIOLOGY-D-20-00029R1 entitled “Human MAIT cell cytolytic effector proteins synergize to overcome carbapenem resistance in *Escherichia coli*" by Boulouis *et al.* for publication in PLOS Biology. We would like to thank the reviewers for their positive comments and constructive criticisms that have helped us further improve the manuscript. With these improvements, we hope that you will find this manuscript suitable for publication.

Point-by-point reply to the specific comments raised by the reviewers is listed below. The changes we made to the manuscript text in response to reviewers’ comments are highlighted in yellow.

**Editor’s comment:** “In addition to the concerns raised by the reviewers, our Academic Editor would like you to clarify whether the E coli you are studying are inside or outside the cells in each assay. If you think the bacteria are sometimes intracellular and sometimes extracellular, please provide a discussion on whether the action of MAIT cells is different in the two cases. We cannot make any decision about publication until we have seen the revised manuscript and your response to the reviewers' comments. Your revised manuscript is also likely to be sent for further evaluation by the reviewers.”

**Response:** Thank you for the possibility to submit a revised version of our manuscript to PLOS Biology. Our study is divided into two main parts. In the first part, we examined the mechanism underlying MAIT cell antimicrobial activity against cell-associated (i.e. in intracellular compartments) *E. coli*. We found that the primary mechanism by which MAIT cells kill cell-associated *E. coli* (incl. carbapenem-resistant strains) is through the release and activity of cytolytic proteins inside infected cells, namely that of granulysin (Gnly) and granzyme (Grz) B. These results are presented in Fig. 1-3 and fig. S1-S3. In the second part of the study, we discovered that a very similar antimicrobial mechanism of MAIT cells (i.e. Gnly and GrzB activity) is also acting on free-living extracellular *E. coli*. Moreover, we found that Gnly and GrzB-dependent MAIT cell antimicrobial activity can enhance carbapenem activity and overcome carbapenem resistance in free-living *E. coli*. These results are presented in Fig. 4-5 and fig. S4-S7. We thus propose a model in Fig. 6 where the same mechanism of MAIT cell antimicrobial activity is acting on both cell-associated and free-living extracellular *E. coli*.

We thank the Academic Editor for pointing out this potential confusion. We have now clarified and discussed this aspect in the manuscript. We further believe we have addressed all concerns raised by the reviewers, as detailed below.

**Reviewer 1**

**General comments:** “The authors have shown that MAIT cells mediate MR1-restricted antimicrobial activity against clinical E. coli isolates in a manner dependent on the activity of cytolytic proteins, but independent of production of pro-inflammatory cytokines or induction of apoptosis in infected cells. The work has been well-planned and executed and is aimed at expanding the salubrious roles of MAIT cells across to clinical microbiology.”

**Response:** We thank the reviewer for the overall positive assessment of our manuscript.

**Specific comment 1:** “What was the volume of blood drawn from donors? The purity of MAIT cells is a key concern as 30% of ancilliary cells could still play a role in overcoming MAIT cell functions or at least impacting the outcome of the findings, for instance sourced from IFNγ, TNF, and IL-17A, pro-inflammatory cytokines. The authors must ensure to improve the purity of MAIT cells used in the experiments. Further, did the authors conduct any baseline investigations on the donors for recruiting into the study? There must be some inclusion and exclusion criteria before recruiting into the study.”

**Response:** Peripheral blood was collected from healthy blood donors recruited at the apheresis unit, Bloodbank@HSA, Health Services Authority, Singapore. The original volume of blood drawn from these donors ranged from 350 to 450 mL. Following apheresis, the volume of blood containing concentrated leukocytes was approximately 25 mL.

We assumed Reviewer 1 referred the 30% of ancillary cells as the residual Vα7.2^neg^/MR1-5-OP-RU^neg^ non-MAIT T cells remaining in the culture after 15-17 days of MAIT cell expansion using the 5-OP-RU and IL-2+IL-7 stimulation (fig. S1A). Although the minimum MAIT cell purity for our antimicrobial assay was 70% as stated in the Materials and Methods section, the median purity of MAIT cells used in our assays was ca. 87%. To reflect our typical MAIT cell purity better, we have now replaced original MAIT cell FACS plots before and after expansion with a more representative plot, and include a new dataset on MAIT cell purity following expansion *in vitro* as a panel in fig. S1A.

Importantly, the residual non-MAIT T cells did not degranulate at an appreciable level following stimulation with *E. coli*-infected HeLa cells (RFig. 1A). Furthermore, in selected experiments shown in fig. S1I-K, we FACS-sorted MAIT cells and the residual non-MAIT T cells from the *in vitro* expansion culture, so that both MAIT and non-MAIT T cells reached a purity of >95% (RFig. 1B). In these experiments, MAIT cells mediated cytotoxicity against infected cells and antimicrobial activity against cell-associated *E. coli*, whereas the non-MAIT T cells did not (fig. S1I-K; RFig. 1C). Non-MAIT T cells also produced far less pro-inflammatory cytokines (RFig. 1D). For the antimicrobial activity of the MAIT cell secretome, we further purified the MAIT cells from the expansion culture to >95% purity for each experiment (fig. S4A). Taken together, we are confident that the antimicrobial activity observed in our study is directly derived from the MAIT cell population.

RFig. 1. **(A)** Degranulation by MAIT and MR1-5-OP-RU^neg^ non-MAIT T cells following co-culture with *E. coli*-infected HeLa cells. **(B)** Purity of FACS-sorted MAIT cells and MR1-5-OP-RU^neg^ non-MAIT T cells. **(C)** Killing of *E. coli*-infected cells by and degranulation of MAIT cells and MR1-5-OP-RU^neg^ non-MAIT T cells. **(D)** Pro-inflammatory cytokine production by MAIT cells and MR1-5-OP-RU^neg^ non-MAIT T cells.

The current study does not utilise blood samples from any clinical trial. The buffy coats were from healthy, anonymous donors recruited at the apheresis unit, Bloodbank@HSA, Health Services Authority (HSA), Singapore. The donors have been tested for and free from HBV, HCV, HIV-1, HIV-2, syphilis, and ZKV. Please refer to the HSA website (<https://www.hsa.gov.sg/blood-donation>) for comprehensive inclusion and exclusion criteria for blood donation in Singapore.

**Specific comment 2:** “The authors have not shown any data on MAIT cell activation markers or proof for MAIT proliferation (i.e. Ki67).”

**Response:** We thank the reviewer for pointing this out. In our MAIT cell expansion culture, we assessed proliferation in selected experiments using Cell Trace Violet (CTV) dilution assay on day 6. In all cases, MAIT cells proliferated vigorously following stimulation with 5-OP-RU and IL-2+IL-7, whereas non-MAIT T cells did not. We did not assess proliferation beyond day 5-6 using the CTV dilution protocol, as the CTV dye would have been diluted too much, rendering it unresolvable from the background fluorescence. We have now included this data in fig. S1A.

With regard to the MAIT cell-killing of infected cells, the assay was performed for only 3 h, and thus there was insufficient time for MAIT cells to proliferate. We assessed MAIT cell activation in these experiments using the MAIT cell degranulation/exocytosis marker CD107a as shown in Fig. 1A.

**Specific comment 3:** “Basic microbiological data on the isolates of E.coli tested may be provided as supplementary data, and the authors should have used a carbapenem sensitive E.coli to prove the beneficial effect of MAIT cells on carbapenem-resistant E.coli. How do the authors justify carbapenem resistance in the isolate tested?”

**Response:** The microbiological data on the *E. coli* isolates are shown in Tables S1 and S2. Carbapenem-sensitive *E. coli* was used in the first part of the study (Fig. 1 and 2). Carbapenem resistance was tested using protocols detailed in the Supplementary Material and Methods.

**Reviewer 2**

**General comments:** “In this manuscript, Boulouis and colleagues describe a possible mechanism underscoring the antimicrobial activity of MAIT cells, based on the cytolytic proteins granulysin and granzyme B, released by activated MAIT cells in an MR1 dependent manner. The paper is well written, the experiments are well controlled and the data are convincing. This is an important study that extends our understanding of the biological functions of MAIT cells, and suggests way of potentially harnessing these cells for clinical benefit, during antibiotic resistant infections.”

**Response:** We thank the reviewer for appreciating our study and the overall positive assessment of our manuscript.

**Specific comment 1:** “The main limitation of this study is that the antimicrobial effect of MAIT cell supernatants is only observed upon culturing the cells for two weeks, and although this is well discussed and acknowledged, it has not been investigated whether tissue resident MAIT cells (ie in the gastrointestinal tract) would express higher levels of granzyme B and granulysin or whether upon infection, in vivo, the kinetics of GrzB and Gly acquisition are faster.”

**Response:** In the early stages of the project, we cultured MAIT cells for 6-7 days before use in killing assays of *E. coli*-infected cells and/or to make supernatants. However, because the MAIT cell yields were insufficient, we decided to prolong the expansion time to at least 14 days. Nevertheless, we still saw antimicrobial activity of MAIT cell secretome when the supernatants were prepared from MAIT cells expanded for 6-7 days only (see new fig.S4G). Our data strongly indicate that the MAIT cell antimicrobial activity (both cell-mediated and secreted) depends on both Gnly and GrzB. Because Gnly is not expressed by MAIT cells until around 5 days post-activation (new Fig. 1K and L, fig.S2G), we therefore did not routinely assess supernatants for antimicrobial activity from earlier time-points. The reason why MAIT cells express Gnly late following activation is unclear, however, it is widely known that Gnly is also expressed late by activated CD8+ T cells. Indeed, Gnly was first discovered as a late activation marker in T cells (Jongstra et al, J. Exp. Med. 1987, 165:601). Additionally, it is possible that Gnly and GrzB expression would be maintained long-term by MAIT cells following acquisition *in vivo*, thus allowing antigen-primed, Gnly^+^ GrzB^+^ MAIT cells to immediately mount antimicrobial activity following cognate recognition. Consistent with this hypothesis, our preliminary data indicate that MAIT cells maintain both Gnly and GrzB for at least 4 weeks following *in vitro* activation and culture (RFig. 2). We have already discussed the temporal regulation of MAIT cell expression of cytolytic proteins and antimicrobial activity in the original manuscript version. However, we prefer not to include RFig. 2 in the main manuscript as these preliminary findings are outside the scope of the current study.

RFig. 2. Long-term expression of Gnly and GrzB by MAIT cells at indicated time-points following in vitro expansion and culture. Representative histogram from 6 independent donors is shown.

In the current study we did not aim to more broadly investigate the expression of GrzB and Gnly in MAIT cells resident in different tissues, or whether the kinetics of acquisition of GrzB and Gnly following stimulation may be similar or different *in vitro* or infection *in vivo*. While these are very important questions to address, the primary aims of the current study are to understand the mechanism underlying MAIT cell antimicrobial activity, and whether this mechanism extends to MDR-GNB, using CR-*E. coli* as a model pathogen. Thus, we believe this very significant undertaking will essentially need its own dedicated study that falls outside the scope of the current study. For instance, to study GrzB and Gnly expression kinetics in an *in vivo* model of bacterial infection, we will need a transgenic mouse system, as rodents do not express Gnly.

Furthermore, due to the Covid-19 pandemic, Singapore (where the senior author EL managed the majority of this study) has raised the Disease Outbreak Response System Condition (DORSCON) alert level to Orange. The DORSCON Orange alert level bars us and our local collaborators from collecting any new samples, incl. gastrointestinal mucosa tissue samples requested by Reviewer 2. Moreover, the most recent social distancing policy also dictates that all non-Covid-19 research work to temporarily cease, making it practically impossible to perform further experiments. Nevertheless, we addressed parts of this question through an examination of tissue resident MAIT cells derived from previously collected nasopharyngeal mucosa tissue samples.

In this new set of experiments, we measured cytolytic proteins (Gnly, GrzA, GrzB, GrzK, Prf) in matched peripheral blood and nasopharyngeal (NP) mucosa tissues derived from 3 healthy donors undergoing nasal polyp removal. In this limited set of donors, we found that CD69^+^ CD103^+^ tissue-resident MAIT cells obtained from the healthy NP mucosa (new Fig. S2F) overall expressed a similar set of cytolytic proteins, although at somewhat lower proportion and magnitude of cytolytic proteins at baseline and following *in vitro* culture when compared to those of matched PB MAIT cells (new Fig. 1K). Interestingly, the kinetics of cytolytic protein acquisition in NP MAIT cells appear to be comparable to that of PB MAIT cells (new Fig. 1L, new fig. S2G), suggesting that tissue-resident MAIT cells are able to respond to infected cells and mount cytotoxicity against infected cells. Indeed, we found that NP MAIT cells efficiently killed *E. coli* EC120S-infected HeLa cells (new Fig. 1M). Overall, in this limited set of donors, our findings suggest that tissue-resident MAIT cells may play an important role in eliminating bacteria-infected cells within the mucosal tissue environments.

**Minor comment 1:** “The legend of Figure 1 G-J is not clear: the text talks about cells activated by 5OPRU or E coli but this is not clear from the graphs; furthermore, it is not clear how the heat map in panel J is derived: are these FACS data?”

**Response:** HeLa cells were infected with *E. coli* EC120S, then purified MAIT cells from d0, 2, or 15 of culture were co-cultured with infected HeLa cells to determine MAIT cell degranulation (Fig. 1G, fig. S2A), killing of infected HeLa cells (Fig. 1H, fig. S2A), and MAIT cell antimicrobial activity (Fig. 1I). 5-OP-RU-pulsed HeLa cells were used as positive controls for MAIT cell degranulation (fig. S2B) and killing of HeLa cells (fig. 2C). Indeed, the heat map in panel J is derived from FACS data. We have now clarified these aspects in the revised version of the manuscript. We thank the reviewer for pointing this out.

**Minor comment 2:** “Figure S2E is misleading, if compared to S2D: although the colour scale reflects the MFI values, it gives the false impression that some markers are not expressed on D0, which is clearly not the case for Granzyme A and perforin. Why was Granzyme K not measured?”

**Response:** We thank the reviewer for pointing this out. We have changed fig. S2E to a graph representing true MFI values for each cytotoxic protein to better present our data. Early in this study, we did not include GrzK in our experiments due to the lack of defined roles of GrzK in cellular cytotoxicity and antimicrobial activity. We have now incorporated GrzK into the new fig. S2D and E from another set of experiments. Based on our findings in Fig. 2, we interpret that GrzK does not play any major role in MAIT cell-mediated cytotoxicity and antimicrobial activity in our model.

**Minor comment 3:** “In Figure 1A and S2F it would be good to have the values of MAIT cell expression of the indicated proteins shown as additional overlay. Also, the authors should show the gating strategy used to discriminate between Hela and MAIT cells.”

**Response:** We believe that the reviewer meant Fig. 2A instead of Fig. 1A, as we have already shown the expression of GrzB and Gnly on the right hand FACS plot subfigure in Fig. 1A (right next to Fig. 1B). Thus, we have now included the MAIT cell cytolytic protein expression overlay in new Fig. 2A and new fig. S2H (original fig. S2F).

**Minor comment 4: “**In Figure 3A, IL17 secretion by MAIT cells is very low and without negative controls (unpulsed target cells for example), should be interpreted with caution.”

**Response:** The reviewer is correct that IL-17 production by MAIT cells is low. This is a well-known feature of peripheral blood human MAIT cells (in contrast to human tissue-resident MAIT cells) that has been published extensively by our group and others. We always included negative controls in our experiments, but due to an oversight, we did not include the data in the previous version of our manuscript. We thank the reviewer for pointing out this oversight. We have now included these data in the new Fig. 3A and fig. S3J. As seen here, though % IL-17 following 24 h stimulation with CR-*E. coli* is relatively low, it is still far higher than the virtually undetectable background level of the unpulsed negative controls. We also affirm that the interpretation in our original version of the manuscript is correct: “All CREC strains induced degranulation and expression of GrzB, IFNγ, TNF, and IL-17A by MAIT cells at comparable levels (Fig. 3A, fig. S3J).”

**Minor comment 5: “**Figure 3: It is not clear why in panels C-E MR1 overexpressing 293T cells were used. The transition from 293T (panels C-E) to Hela is also confusing. Lastly, what is the difference between panels D and E?”

**Response:** In the early phase of the study, we used the 293T-hMR1 cell line as a target cell line to quickly assess MAIT cell cytotoxic responses against target cells pulsed with a number of CREC strains. As the study progressed, we wanted to measure MAIT cell antimicrobial activity against a range of *E. coli* strains. To perform the MAIT cell antimicrobial activity assay (modified from gentamicin-protection assay), we needed an adherent cell line. 293T-hMR1 cells are a semi-adherent cell line, which led to cell loss during the multiple washing steps. Therefore, we opted to use HeLa cells as adherent target cells (and A549 cells in selected experiments per fig. S1F-H). These cell lines have the added advantage that they express an endogenous level of MR1, instead of the MR1-overexpression of 293T-hMR1 cell line.

We agree with the reviewer that this could be confusing. Thus, we performed a new set of experiments where we assessed MAIT cell killing of HeLa cells pulsed with drug-sensitive *E. coli* EC120S or the CR-*E. coli* strains EC234, EC241, and EC362 (n=5 independent donors for each strain). We also reduced the MAIT cell-HeLa cell co-culture to 3 h to be consistent with the rest of the manuscript (instead of the 24 h co-culture in the original Fig. 3C-E). The results were similar to those of MAIT cell killing of 293T-hMR1 cells pulsed with the same bacterial strains. The conclusion has therefore remained unchanged, i.e. MAIT cells can recognise and effectively kill CR-*E. coli*-pulsed target cells. To avoid confusion, we have now replaced the original Fig. 3C-E with the data from this new set of experiments using *E. coli*-pulsed HeLa cells. The original Fig. 3C-E were moved as a new fig. S3M-O.

The difference between old panels D and E is that panel D shows early apoptosis (Casp3 positive but negative for amine-reactive Dead Cell Marker (DCM)), whereas panel E shows late apoptosis (Casp3+ DCM+). Given in the new Fig. 3D we only did the co-culture for 3 h, and thus no late apoptosis events were observed at this point in time (see Fig. 1B), we now only show Casp3+ DCM- in the new Fig. 3D. The original Fig. 3F and G have now become the new Fig. 3E and F, respectively.

Collectively, the revisions done in the new Fig. 3 should now clarify the message and prevent confusion.

**Minor comment 6:**  ”Figure 4: in panel D, at 2 hours, the authors detect a significant difference in bacterial counts, upon exposure to activated MAIT supernatants. This, however, is not reflected in the time curves in panels G, H and in Figure S5A and C.”

**Response:** MAIT cell secretomes can directly damage bacterial membrane integrity as shown in Fig. 4B and C, which in turn leads to significant bacterial growth suppression, as shown in Fig. 4D. This significant bacterial membrane damage and bacterial growth suppression, however, is considered mild, given we observed less than half of the bacterial population stained positive for Sytox Green (Fig. 4C), and a reduction of 1 log_10_ or less in bacterial counts after 2 h of incubation (Fig. 4D), respectively. It was then not too surprising that we did not see a reflection of this mild but significant inhibition in the time-kill analyses shown in Fig. 4G and H, in the time curves in Fig. S5A, and at 18 h post-incubation in Fig. S5C. Probably, the majority of surviving bacteria continued to grow over the 18-24 h period. Only when we combined MAIT cell secretomes with carbapenems did we see a prolonged bactericidal activity (that was not seen in either treatment alone).

The observation that MAIT cell secretomes alone were not able to completely damage the bacteria and suppress the growth could potentially be explained by short biological activity half-lives of the cytolytic proteins (ie. GrzB and Gnly) within the secretomes. The experimental design included no fresh inputs of MAIT cell secretomes during culture. There were also no living MAIT cells present to continuously secrete and “replenish” Gnly and GrzB into the secretomes. To address this potential explanation, we would need a dedicated future study employing a dynamic culture system, such as through a hollow-fibre infection model that could mimic the pharmacokinetic properties of secreted GrzB and Gnly. Such a study is out of scope of the current manuscript.

An indirect way to partially address this issue is to reduce the original inoculum number. In all cases, we used a starting inoculum of 5 log_10_ CFU/mL. The reason we used such a high starting inoculum was to adhere to the Clinical and Laboratory Standards Institute (CLSI) and European Committee on Antimicrobial Susceptibility Testing (EUCAST) standards in testing for antimicrobial susceptibility, and additionally to test if MAIT cell secretomes can successfully enhance carbapenem bactericidal activity and overcome carbapenem resistance at high bacterial loads. Indeed, when we serially diluted the starting inoculum, we saw a reduction of the bacterial loads by the MAIT cell secretomes alone (in the absence of carbapenems) that is reflected in the time-kill analyses. We have now included these new results in the new Fig. S4G and added aspects of the biological activity of cytolytic proteins in MAIT cell secretomes and inoculum effect on MAIT cell antimicrobial activity in the Discussion section.

**Minor comment 7: “**The text mentions (page 14) expression of GrzA and Prf in figure 5I, but this is only shown in S7A and B.”

**Response:** The text meant to state that GrzA and Prf could not be detected inside bacterial cells, as shown in fig. S7A and B, not in Fig. 5I. We have now deleted Fig. 5I reference from the text. We thank the reviewer for pointing out this typo.

**Minor comment 8:** ”Figure S1A, is the y axis correct? Should it not be % of CD107+ cells, without MAIT?”

**Response:** In Figure S1A, the y-axis of the plots is correct. However, we modified the y-axis title in the original fig. S1I (new fig. S1J) where it should read “CD107a+ (% of cells)”. We thank the reviewer for pointing out this typo.

**Minor comment 9:** ”Figure S3K, the colour legend is missing.”

**Response:** We thank the reviewer for spotting this mistake. We have now added the colour legend for Figure S3K.

**Minor comment 10:** “Colours in the growth curves (S5A, S6K) are often difficult to distinguish, perhaps a thicker line could be helpful.”

**Response:** We have changed the line thickness in order to help readers distinguish the growth curves.

**Minor comment 11**:  ”Why the supernatant of 293T and 5OPRU (but no MAIT cells) has an anti-bacterial effect in Fig S5F?”

**Response:** All 293T+5-OP-RU control supernatants had a transient antibacterial effect when imipenem was added incl. Fig. 4G & H (solid line of respective colours) and fig. S5F. However, there was never a sustained bactericidal effect (>3 Log_10_ reduction in bacterial loads over 24 h period), as strains EC234 and EC362 are resistant to imipenem due to their expression of carbapenemases. The initial drop was likely due to the presence of susceptible minority subpopulations within the bacterial cultures when we sub-cultured these strains overnight in the absence of any antibiotics for all functional assays. When exposed to imipenem added in 293T+ 5-OP-RU control supernatants, these less resistant subpopulations were probably quickly killed and the highly resistant subpopulations took over and grew. In any case, the overall take home message is that no or very little growth was detected when imipenem was added into MAIT cell supernatants instead of 293T+ 5-OP-RU control supernatants. This means that even the highly resistant populations were completely killed by the combination actions of MAIT cell secretomes and imipenem.

**Minor comment 12**: ”The dual labelling of bacteria with Sytox green and Syto62 is very elegant, but from the text I had the impression bacteria were labelled with Syto62 before exposure to MAIT supernatants and carbapenem, hence it was not clear why MFI of Syto62 was also increasing. This was clear after reading the methods, so it would help if the main text or the legend also explained the experiment.”

**Response:** We thank the reviewer for pointing this out. We have now added an explanation in the main text to avoid any misunderstanding.

**Reviewer 3**

**General comment:** “This paper entitled "Human MAIT cell cytolytic effector proteins synergize to overcome carbapenem resistance in Escherichia coli" by Boulouis and colleagues presents results to address the mechanisms behind the antimicrobial activity of circulating human MAIT cells and explores the ability of MAIT cells to target multidrug resistant E coli and the mechanism  that may be mediating this activity.

Given that MAIT cells are notably found to be much more sensitive to several bacterial infections there is considerable interest in understanding the mechanisms that drive MAIT cell activity. This study demonstrates key effector pathways (granulysin and Grnazyme B, that are involved in MAIT cell activity against both cell associated and free living drug sensitive and resistant bacteria (in this case E Coli strains).

The study is a well-designed study testing several mechanisms behind human peripheral MAIT cells antimicrobial activity. The authors have evaluated key molecular pathways and include numerous controls, in comparison to non MAIT cells and pharmacological inhibitors to determine the precise role of various lytic mediators in MAIT cell activity. The report presents very relevant findings particularly highlighting the unique importance of granulysin (which has been unclear - see PLoS Pathog. 2013 Oct; 9(10): e1003681.) using a variety of assays. The approach to address the mechanism of MAIT cells activity against carbapenem AMR bacteria is intriguing and novel. Overall the manuscript has well presented data however suggestions for improvement should be considered.

A very detailed assessment of MAIT cells antibacterial activity is presented by the authors and the results are interesting. The growing threat to public health includes the rapid spread of CRE into the community and the provocative results on the role MAIT cells play in disarming carbapenem activity is a future option to be considered based on the data presented. Limitations are somewhat acknowledged as in vivo studies are lacking to validate some of the findings and this can be enhanced in the manuscript. Nevertheless, the authors reveal interesting findings on MAIT cell antimicrobial activity with clinical relevance and well-presented model in Fig 6.”

**Response:** We thank the reviewer for the overall very positive assessment of the manuscript. We have further enhanced the discussion regarding potential *in vivo* studies for data validation.

**Specific comment 1:** “Previous studies have shown that MAIT cell require a cytoliytic effectors and utilize the granzyme mediated pathways for killing (Kurioka et al Mucosal Immunol. 2015;8(2):429-40. So it is unclear on the novel findings presented in this report.”

**Response:** The study by Paul Klenerman’s group (Kurioka et al, Mucosal Immunol. 2015), as well as from Olivier Lantz (Le Bourhis et al PLOS Pathog. 2013), and our lab (Leeansyah et al PLOS Pathog. 2015), collectively show that MAIT cells can kill infected cells through the release of cytolytic proteins. However, none of these studies explored the mechanisms underlying MAIT cell direct antimicrobial activity against bacteria. In this study, we show that cytolytic proteins mediate antimicrobial activity by MAIT cells, particularly through the combined effects of Gnly and GrzB. We additionally show that the same antimicrobial mechanism extends to control of carbapenemem-resistant *E. coli*. Importantly, our results show that cytolytic protein-dependent MAIT cell antimicrobial activity can overcome carbapenem resistance and enhance carbapenem bactericidal activity against multidrug resistant strains of *E. coli*.. Collectively, the current study presents novel and highly clinically relevant findings on how MAIT cell antimicrobial activity works, and can overcome carbapenem resistance in *E. coli*.

**Specific comment 2:** ”The mechanism underlying antibacterial MAIT cell killing is comprehensive however the role of FAS/FASL or other contact dependent pathways was not determined. Co-culture transwell studies would clarify this further.”

**Response:** The involvement of Fas/FasL or contact dependent killing was not directly assessed in this study. A previous study by Kurioka et al Mucosal Immunol. 2015 (Fig. 3E) found that MAIT cell killing was independent of the Fas/FasL pathway. Nevertheless, we revisited our MAIT cell secretome data and did not detect a significant increase in sFas or sFasL in the supernatant of *E. coli*-infected HeLa cells following co-culture with MAIT cells in (RFig. 3), in agreement with the Kurioka et al Mucosal Immunol. 2015 study. Moreover, our data clearly indicate that the mechanism of MAIT cell killing of infected cells and subsequent antimicrobial activity of cell-associated *E. coli* is predominantly dependent on MR1-TCR interaction (Fig. 1C-E; Fig. 3E and F), as well as exocytosis of cytolytic proteins (Fig. 2I-L). Considering these findings, we believe that the Fas/FasL pathway is not significantly involved in the killing of infected cells in our *in vitro* model. Furthermore, our findings of MR1-dependency and cytolytic protein-mediated killing of infected cells also strongly indicate that cell-to-cell contact is required to mediate killing and antimicrobial activity in our model. Therefore, we believe that co-culture transwell studies would not add further significant value to the current study. Nonetheless, the Reviewer is prudent to point out that other cell-to-cell contact-dependent mechanisms may play an additional role in mediating killing of infected cells and/or antimicrobial activity. Such studies, however, will be extensive and require their own dedicated projects in the future.

RFig. 3. Levels of sFasL and sFas in the supernatants of MAIT cell and HeLa cell coculture in the absence of presence of E. coli EC120S. ns, not significant.

**Specific comment 3: “**Fig 1G - would polyclonal stimulation rather that cytokine driven MAIT cell activation control bacterial loads?”

**Response:** Early in the course of the present project, we indeed activated and expanded purified MAIT cells polyclonally using the ImmunoCult CD3/CD28/CD3 T cell activator (STEMCELL Technologies) supplemented with IL-7, as described in the main Materials and Methods section. As the project progressed, we decided to activate and expand MAIT cells using the 5-OP-RU+IL-2+IL-7 protocol as this gave us much higher MAIT cell yields needed to perform a multitude of functional assays. We saw similar functional performance by MAIT cells expanded by using these two protocols. For example, killing of infected cells by MAIT cells was comparable to that of MAIT cells expanded using the 5-OP-RU+IL-2+IL-7 protocol, as shown in the original Fig. 3C-E (now new fig. S3M-O). Additionally, control of free-living bacterial growth by polyclonally-activated MAIT cell secretomes was also comparable to that of MAIT cell secretomes derived from the 5-OP-RU+IL-2+IL-7 protocol, as shown by the new fig. S4G.

Unless otherwise specified, MAIT cell activation and expansion *in vitro* was done using the 5-OP-RU+IL-2+IL-7 protocol. We have now clarified this aspect in the figure legends.

**Specific comment 4:** ”Unclear in Fig 2 if non MAIT cells were tested as controls”

**Response:** In Fig. 2, non-MAIT cells were not tested as controls. However, non-MAIT cells were shown to neither effectively degranulate, kill infected cells, nor control the bacterial loads shown in the original fig. S1H-J (new fig. S1I-K). Therefore, further investigations using non-MAIT cell controls were not pursued in Fig. 2.

**Specific comment 5:** Figure 5 D and F are not convincing and could be a floor effect.

**Response:** In our experiments, CR-*E. coli* strain EC362 appeared to be more susceptible to the antimicrobial effects of combined imipenem+MAIT cell secretomes. In our time-kill analyses where we used MAIT cell supernatants combined with imipenem concentrations equivalent to 50% (4 μg/mL) or 100% (8 μg/mL) of EC362’s MIC, the bacteria were quickly killed and their numbers remained undetectable in half or all of the experimental groups after 24 h, respectively (Fig. 4H). Unfortunately, due to the labour-intensive nature of these experiments, we were unable to perform our time-kill analyses using titrated imipenem concentrations possibly avoiding such complete inhibition, and allowing better data spreads for correlative analyses. Nevertheless, we chose the MAIT cell secretome + 4 μg/mL imipenem experimental dataset to investigate possible associations between MAIT cell secretome antimicrobial activity with MAIT cell-derived cytolytic proteins.

In these correlative analyses, we found significant negative correlations between surviving bacterial loads and Gnly or GrzB levels in MAIT cell secretomes (Fig. 4D and F). No such correlation was seen with GrzA or Prf levels (Fig. S6B and D). The same correlation patterns were also found when another CR-*E. coli* strain (EC234) was used (Fig. 5C and E). In yet another analysis, we noted that the EC362 experimental condition with no detectable bacterial growth after 24 h incubation with MAIT cell supernatants + 4 μg/mL imipenem had significantly more Gnly and GrzB in the MAIT cell secretomes, but again no difference in GrzA and Prf levels (Fig. 5B). When we subsequently analysed the MAIT cell secretome Gnly levels dataset into quartiles, supernatants that contained the highest concentrations of Gnly had significantly greater and longer inhibition on EC362’s growth (fig. S6G).

Therefore, although the bacterial loads from half of the experimental group were undetectable and suggested by the Reviewer to contribute to the floor effect of our correlative analyses shown in Fig. 5D and F, collectively the aforementioned data analyses argue against this. Particularly, the observations that: 1) no correlation was observed between bacterial loads and the levels of GrzA and Prf in MAIT cell secretomes of the same experiments (fig. S6B and D), 2) the same correlation patterns were observed in CR-*E. coli* strain EC234 where there were wide data distributions, 3) the experimental group with no detectable bacterial growth after 24 h incubation with MAIT cell supernatants + imipenem had significantly more Gnly and GrzB in the MAIT cell secretomes, but no difference in GrzA and Prf levels, and finally, 4) MAIT cell secretomes that contained the highest concentrations of Gnly had significantly greater and longer inhibition on bacterial growth, convinced us that the association between Gnly and GrzB levels in the MAIT secretomes with antimicrobial activity remains valid. Indeed, the results shown in Fig. 6G-K that both Gnly and GrzB were detected inside *E. coli* cells and contributed to bacterial damage following incubation with MAIT cell supernatants strongly supports this interpretation.

**Minor comment 1: “**Fig 1 H. HELA/MAIT cells. Some of the descriptions are missing for the - - + third line data.”

**Response:** We thank the reviewer for pointing this out. We believe the reviewer was referring to the original Fig. 2H since the original Fig. 1H (now new Fig. 1G) does not require further description. We added the missing description to the original Fig. 2H.

**Minor comment 2: “**Fig 3A the color shading box description should be included as in Fig 3B”

**Response:** We have added the colour shading box descriptions to both Fig. 3A and B.
