# Supplementary material for: Human MAIT cell cytolytic effector proteins synergize to overcome carbapenem resistance in Escherichia coli
Source: PLoS Biol. 2020 Jun 8;18(6):e3000644. doi: 10.1371/journal.pbio.3000644 (PMC7302869; doi:10.1371/journal.pbio.3000644)
Supplement: Attachment — Submitted filename: Rebuttal letter.docx [file pbio.3000644.s015.docx]

Dear Dr. Di Jiang,

We are delighted that both you and the Academic Editor have considered our revised version of our manuscript PBIOLOGY-D-20-00029R2 entitled “**Human MAIT cell cytolytic effector proteins synergize to overcome carbapenem resistance in *Escherichia coli***” suitable for publication in PLOS Biology as a primary research article.

Point-by-point reply to the specific comments raised by the Editor is listed below. The changes we made to the manuscript text in response to Editor’s comments are highlighted in track-changes. We have addressed all the Editor’s concerns, and with these improvements, we hope that you will find this manuscript is formally acceptable for publication.

**Editor’s comment 1:**

Please create a separate subsection of Ethics Statement and places it in the beginning of the Methods section. Please make sure it includes all relevant information described below.

Please include the full name of the IACUC/ethics committee that reviewed and approved the animal care and use protocol/permit/project license. Please also include an approval number.

Please include the specific national or international regulations/guidelines to which your animal care and use protocol adhered. Please note that institutional or accreditation organization guidelines (such as AAALAC) do not meet this requirement.

Please include information about the form of consent (written/oral) given for research involving human participants. All research involving human participants must have been approved by the authors' Institutional Review Board (IRB) or an equivalent committee, and all clinical investigation must have been conducted according to the principles expressed in the Declaration of Helsinki.

**Response:** As requested by the Editor, in the minor revised version of the manuscript we have added the Ethics Statement in the beginning of the Methods section containing the details of the Institutional Review Board committees and the approval numbers pertaining to the use of human samples. Written informed consent was obtained from all donors, and human samples were collected in accordance to approved study protocols that conform to the Declaration of Helsinki. Please note that the current study does not perform research on animals.

**Editor’s comment 2:**

1) Supplementary files (e.g., excel). Please ensure that all data files are uploaded as 'Supporting Information' and are invariably referred to (in the manuscript, figure legends, and the Description field when uploading your files) using the following format verbatim: S1 Data, S2 Data, etc. Multiple panels of a single or even several figures can be included as multiple sheets in one excel file that is saved using exactly the following convention: S1_Data.xlsx (using an underscore).

2) Deposition in a publicly available repository. Please also provide the accession code or a reviewer link so that we may view your data before publication.

Regardless of the method selected, please ensure that you provide the individual numerical values that underlie the summary data displayed in the following figure panels as they are essential for readers to assess your analysis and to reproduce it: Figs 1B-L, 2B-HJ-L, 3ABD-F, 4ACDF-H, 5A-FHK, S1EH-K, S2BCEGI,S3A-IKNO, S4CF-HK, S5A-DFH, S6A-GI-L. NOTE: the numerical data provided should include all replicates AND the way in which the plotted mean and errors were derived (it should not present only the mean/average values).

Please also ensure that figure legends in your manuscript include information on where the underlying data can be found, and ensure your supplemental data file/s has a legend.

**Response:**

We have included the individual numerical values that underlie the summary data displayed in the figures and supplementary figures into one single Excel spreadsheet file named S1_Data.xlsx as requested. We have added the description on how the plotted means/medians and error bars were derived in each Figure legend. All the main and supplementary figure legends have now also included a statement where the underlying data can be found.

**Editor’s comment 3:**

Please provide an editor/reviewer key/token for Bioproject PRJNA577535 so we can check it before acceptance.

Please ensure that your Data Statement in the submission system accurately describes where your data can be found.

**Response:**

We have provided the publicly available database links on the cover letter. We have also updated the data availability statement in both our manuscript and submission system to accurately describes where the data can be found.
